# Identification of feature autophagy-related genes in patients with acute myocardial infarction based on bioinformatics analyses

**DOI:** 10.1042/BSR20200790

**Published:** 2020-07-09

**Authors:** Yajuan Du, Enfa Zhao, Yushun Zhang

**Affiliations:** Department of Structural Heart Disease, The First Affiliated Hospital of Xi'an Jiaotong University, Xi’an 710061, China

**Keywords:** acute myocardial infarction, autophagy-related genes, database, diagnosis, recursive feature elimination, support vector machine

## Abstract

**Objective:** To identify feature autophagy-related genes (ARGs) in patients with acute myocardial infarction (AMI) and further investigate their value in the diagnosis of AMI.

**Methods:** Gene microarray expression data of AMI peripheral blood samples were downloaded from the GSE66360 dataset. The data were randomly classified into a discovery cohort (21 AMI patients and 22 healthy controls) and a validation cohort (28 AMI patients and 28 healthy controls). Differentially expressed ARGs between patients with AMI and healthy controls in the discovery cohort were identified using a statistical software package. Feature ARGs were screened based on support vector machine-recursive feature elimination (SVM-RFE), and an SVM classifier was constructed. Receiver operating characteristic (ROC) analysis was used to investigate the predictive value of the classifier, which was further verified in an independent external cohort.

**Results:** A total of seven genes were identified based on SVM-RFE. The SVM classifier had an excellent discrimination ability in both the discovery cohort (area under the curve [AUC] = 0.968) and the validation cohort (AUC = 0.992), which was further confirmed in the GSE48060 dataset (AUC = 0.963). Furthermore, the SVM classifier showed outstanding discrimination between AMI patients with and without recurrent events in the independent external cohort (AUC = 0.992). The identified genes are mainly involved in the cellular response to autophagy, macroautophagy, apoptosis, and the FoxO signaling pathway.

**Conclusion:** Our study identified feature ARGs and indicated their potential roles in AMI diagnosis to improve our understanding of the molecular mechanism underlying the occurrence of AMI.

## Introduction

Acute myocardial infarction (AMI), also known as acute heart attack in a clinical background of acute myocardial ischemia, is induced by the sudden blockade or occlusion of a major coronary artery branch, resulting in ischemia or infarction of cardiomyocytes [1]. It is estimated that there were approximately 7.29 million AMIs occurred worldwide in 2015, which contributed to high morbidity and mortality in terms of global health [2]. The current diagnostic approaches for the evaluation of AMI generally comprise a physical examination, a surface electrocardiogram (ECG), and blood testing for gold standard cardiac biomarkers, such as cTnI/T and CKMB. However, these biomarkers have been criticized for their lack of sufficient specificity and sensitivity [3]. Classic risk factors, such as smoking, hypertension, high serum cholesterol, hypercholesterolemia, diabetes mellitus, and obesity, fail to fully explain the risks of morbidity and mortality from AMI. Furthermore, numerous patients present an inexplicable etiology of myocardial damage [4]. Previous studies have reported that patients with coronary heart disease often lack common risk factors; in fact, more than 20% of patients have no commonly accepted risk factors and 40% have only one [5,6]. This evidence indicates that genetic factors may also play an important role in the development of AMI.

Autophagy (meaning “self-eating”), a common phenomenon in eukaryotic cells, is a crucial metabolic process for the degradation of cytoplasmic proteins and organelles into amino acids and fatty acids for energy production and recycling [7]. Previous research has revealed that autophagy in ischemic cardiomyocytes can provide essential energy for cell survival by clearing disordered organelles or aging proteins [8]. At present, more than 30 kinds of autophagy-related genes (ARGs) are believed to be closely involved in autophagy [9,10]. For example, it has been shown that *NR4A2* knockdown aggravates cardiomyocyte apoptosis while *NR4A2* overexpression ameliorates it. *NR4A2* up-regulation is regarded as an adaptive response to ischemia-induced cardiomyocyte apoptosis [11]. However, the role of ARGs in AMI has not been fully elucidated.

In the present study, we utilized gene expression microarray data from the Gene Expression Omnibus (GEO) database and performed mRNA profiling on two cohorts of AMI patients. Differentially expressed ARG expression profiles were identified in control and AMI samples. The recursive feature elimination (RFE) algorithm for effectively improving classification accuracy was used to select the risk genes among feature ARGs [12,13], which were subsequently applied to construct a SVM classifier for distinguishing between AMI and control samples. The predictive value of the classifier in the diagnosis of AMI in two cohorts was explored using receiver operating characteristic (ROC) analysis and further verified in an independent external cohort.

## Materials and methods

### Patient samples and ARGs

Two RNA sequencing datasets were downloaded from the GEO database. The GSE66360 dataset, which was conducted in circulating endothelial cells (CECs), consisted of a discovery cohort (21 AMI patients and 22 healthy controls) and a validation cohort (28 AMI patients and 28 healthy controls). The GSE48060 dataset, which was conducted in whole blood samples, contained 31 AMI patients and 21 healthy controls. Among the AMI patients, 5 had recurrent events while the remaining 26 were event-free over a 1.5-year follow-up. All datasets were produced using the Affymetrix Human Genome U133 Plus 2.0 Array. A total of 232 ARGs were obtained by searching the Human Autophagy Database http://autophagy.lu/) and had been confirmed in previous studies as involved in the autophagy process [14]. The ARG expression matrix was extracted from the GSE66360 dataset using R statistical software (The R Foundation, Vienna, Austria), and a total of 210 ARG expression values were obtained.

### Differentially expressed ARGs

R statistical software (version 3.5.1; The R Foundation; https://www.r-project.org/) and the Bioconductor linear models for microarray data (LIMMA) package were used to screen the significance analysis of differentially expressed ARGs between AMI and control samples, as well as background correction and normalization between arrays [15]. The Bonferroni method was applied to perform multiple test corrections. The threshold for identification of differentially expressed ARGs was set to a *P*-value < 0.05 and |log2fold change (FC)| ≥ 1.

### Feature gene selection and SVM classifier construction

SVM is a supervised learning model that aims to classify data points by maximizing the distance of a hyperplane for classification and regression analysis with high accuracy [16]. The RFE algorithm uses the coefficients of the weight vector to compute the feature ranking score [13]. RFE used for dimensionality reduction clearly contributes to classification accuracy and yields a better classification performance than many other feature reduction methods [13]. Therefore, to select smaller subsets of the most relevant features, we proposed a model combining SVM and RFE algorithms to search for best parameters for gene selection among all ARGs. To test the predictive value of the identified ARG, a heat map of the genes was clustered using the pheatmap package in R (The R Foundation; https://cran.rproject.org/web/packages/pheatmap/index.html) for all samples in the discovery cohort and subsequently confirmed in the validation cohort. Finally, the optimal feature genes after recursive feature selection were put into an SVM classifier with a radial basis function (RBF) kernel and 5-fold cross-validation to make predictions. Furthermore, the performance of the SVM classifier in discriminating AMI from healthy controls in the discovery and validation cohorts was measured as the area under the curve (AUC). The predictive capacity was further validated in an independent external cohort. Moreover, the SVM classifier was used to test the performance in terms of AMI recurrence and non-recurrence in subsets of the GSE48060 dataset, which consisted of 5 AMI patients with recurrent events and 26 event-free patients.

### Functional enrichment analysis of the identified feature genes

Gene ontology (GO) functional enrichment analysis is widely used in functional studies of large-scale transcriptomic or genomic data and defines the concepts used to describe gene function, including biological process (BP), cellular component (CC), and molecular function (MF). To characterize the functions and pathways of the identified feature genes, GO and Kyoto Encyclopedia of Genes and Genomes (KEGG) pathway enrichment analyses were performed to identify functional and metabolic pathways using the biological tool. The clusterProfiler package (The R Foundation) was used to explore the biological meaning and pathways behind numerous genes [17].

### Statistical analysis

R statistical software (version 3.5.1; The R Foundation; https://www.r-project.org/) was used to perform all the statistical analyses in the present study. The feature ARG selection process was performed using the RFE function in the caret package (The R Foundation; https://cran.r-project.org/web/packages/caret/index.html). The SVM classifier from R package e1071 (The R Foundation) was used for classification analysis of the identified feature genes. A 5-fold cross-validation was utilized to evaluate the performance of the prediction model. ROC analyses were performed using the ROCR package in R (The R Foundation), and the AUCs were calculated to evaluate the predictive ability of the built model [18]. Hierarchical clustering analysis was conducted for the identified feature genes using the pheatmap package in R (The R Foundation) based on the Euclidean distance. *P*-value < 0.05 were considered to indicate a statistically significant difference.

## Results

### Identification of feature ARGs

**Figure 1:**
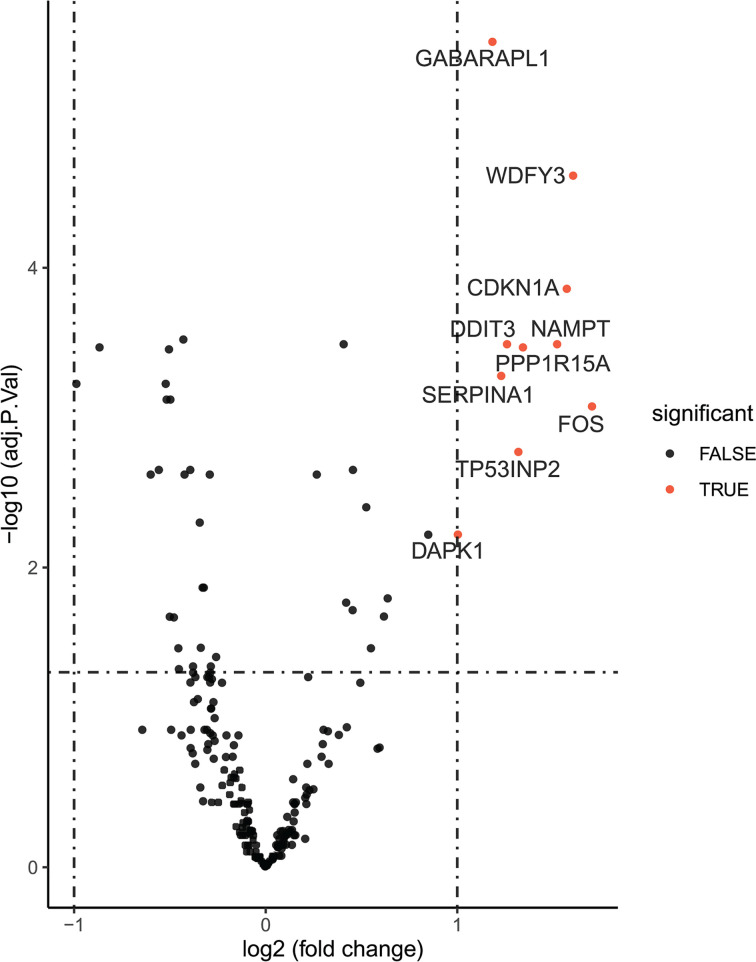
Differential expression of feature autophagy-related genes in acute myocardial infarction tissue samples

**Figure 2:**
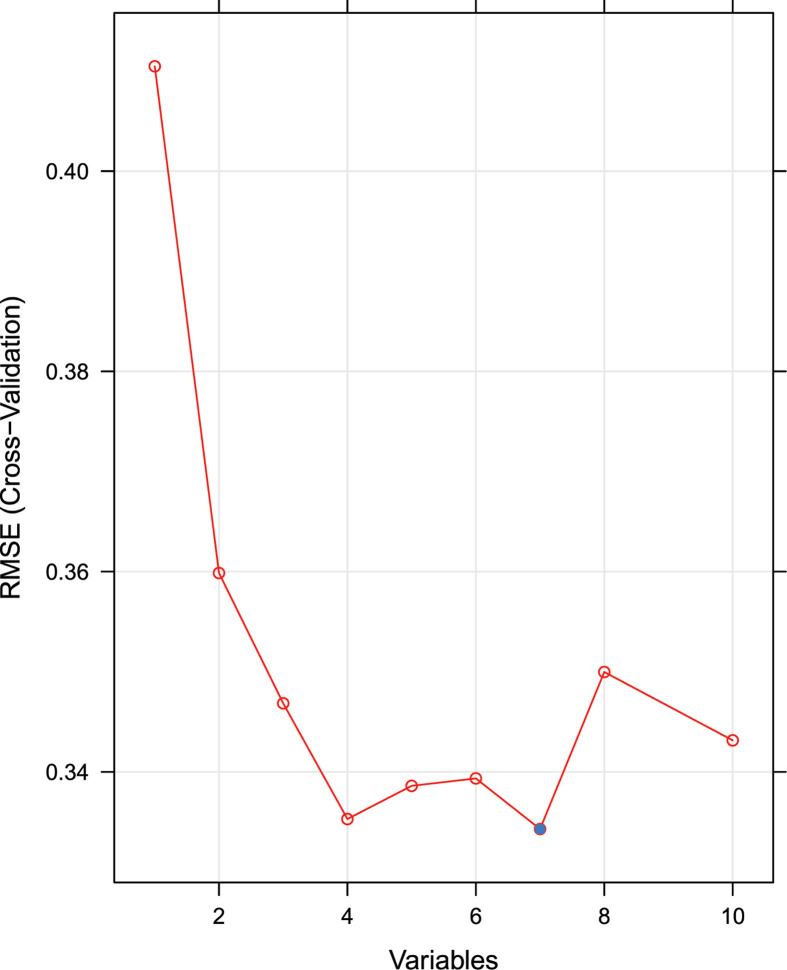
A plot of feature gene selection by recursive feature elimination

**Figure 3:**
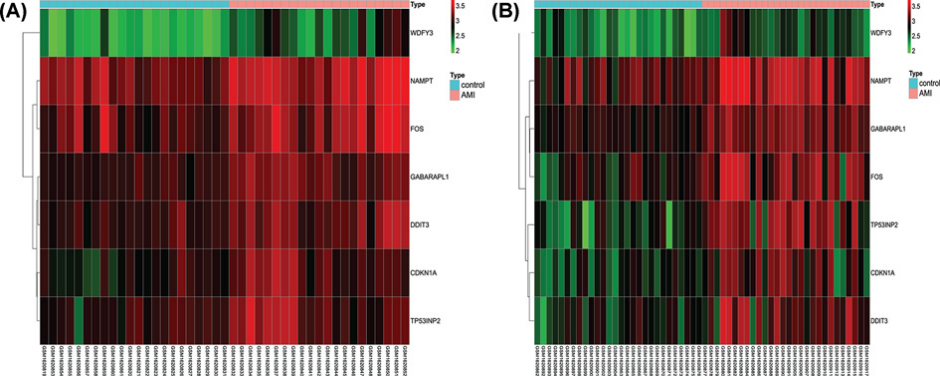
Hierarchical clustering analysis of identified genes Hierarchical clustering analysis demonstrates the identified autophagy-related gene expression patterns in the discovery cohort (**A**) and validation cohort (**B**) in the GSE66360 dataset.

**Figure 4:**
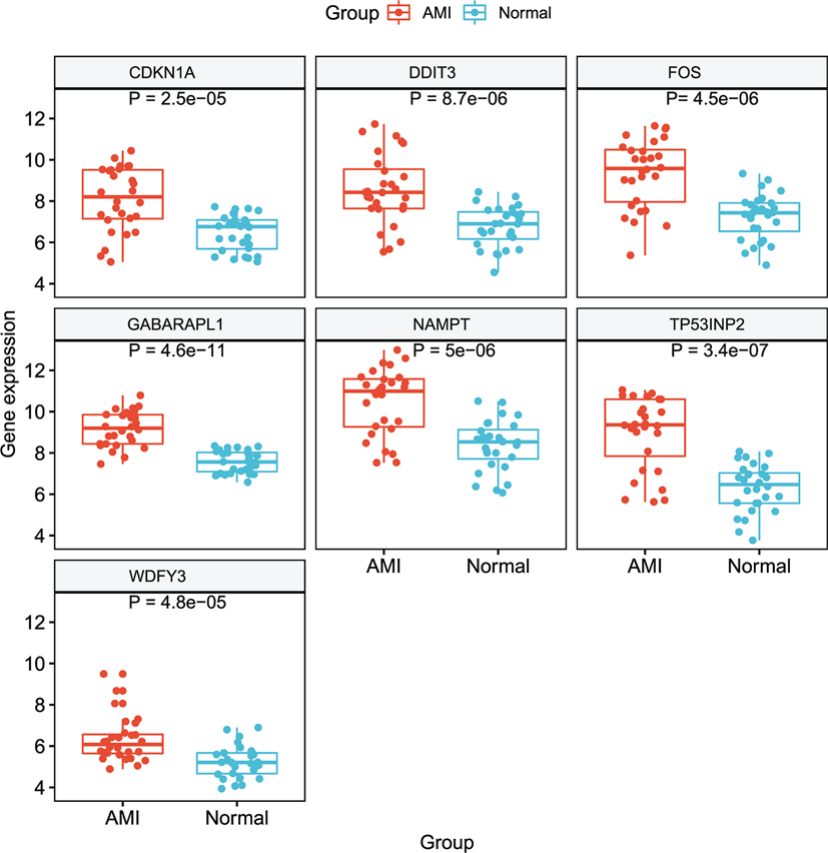
Validation of expression level of identified genes Validation of the expression levels of identified autophagy-related genes in patients with acute myocardial infarction (AMI) in the validation cohort (28 AMI patients and 28 healthy controls).

After background correction and normalization, 10 differentially expressed feature genes were identified among the 210 ARGs. All of the aforementioned feature genes were up-regulated (Figure 1). We used RFE analysis to further screen for the optimal feature genes in the discovery cohort. As shown in Figure 2, using RFE, we were able to reduce the features to seven genes (*WDFY3, TP53INP2, GABARAPL1, CDKN1A, DDIT3, NAMPT*, and *FOS*) with minimum root mean-square error. Hierarchical clustering analysis was carried out for all samples in the discovery cohort according to the seven identified feature genes. As shown in Figure 3A, hierarchical cluster analysis revealed that feature gene expression patterns varied between the AMI and control groups. Hierarchical clustering analysis in the validation cohort also revealed that the patients were clearly separated into two clusters based on the expression levels of the identified feature genes (Figure 3B). To validate the feature ARG expression levels, the identified genes were selected for validation using the validation cohort. As shown in Figure 4, the expression levels of the feature genes in AMI tissue were significantly higher than those in the control group (*P*<0.05). Therefore, the optimal seven feature genes were subsequently utilized to construct the SVM classifier, which was further verified using the independent cohort.

### Diagnostic value of feature ARGs in AMI

**Figure 5:**
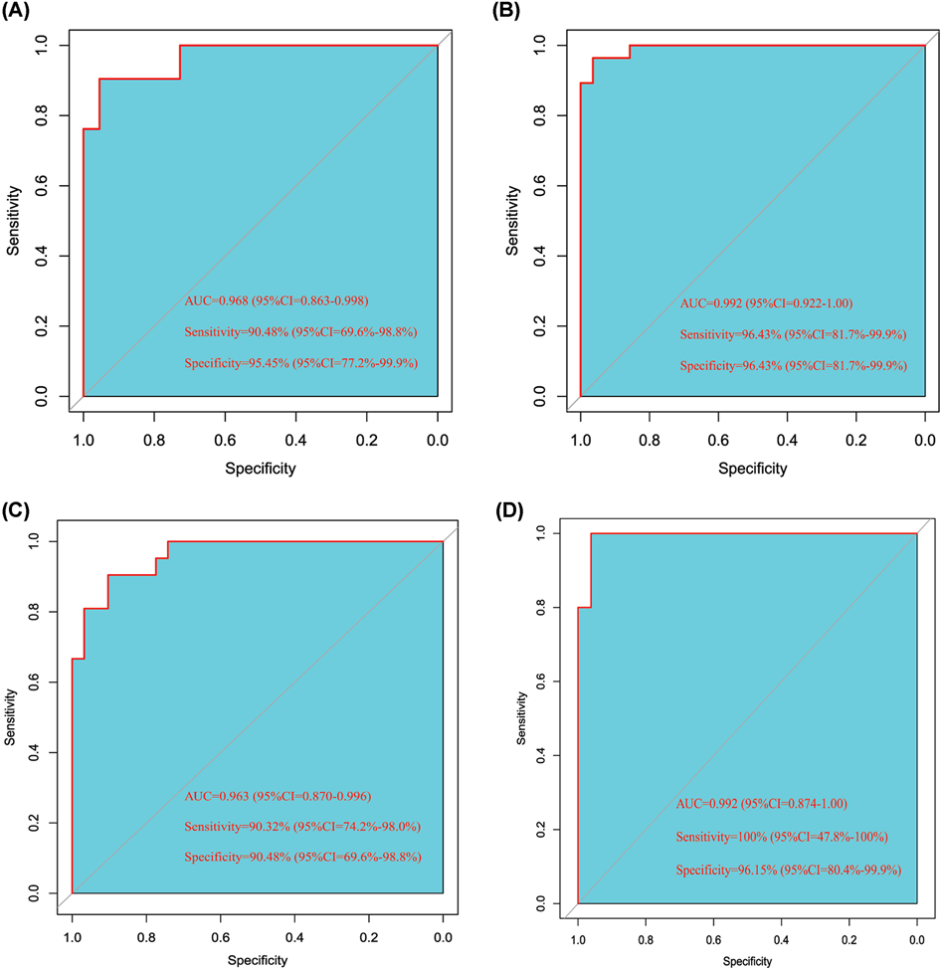
The performance of support vector machine (SVM) classifier Receiver operating characteristic curves of the support vector machine (SVM) classifier for the discovery cohort (**A**) 21 acute myocardial infarction [AMI] patients and 22 healthy controls), the validation cohort (**B**) 28 AMI patients and 28 healthy controls), the independent external cohort (**C**) 31 AMI patients and 21 healthy controls), and recurrent prediction in subsets of the independent external cohort (**D**) 5 recurrent AMI patients and 22 non-recurrent patients).

We further explored the diagnostic value of the feature genes in AMI using SVM with the RBF kernel method and 5-fold cross-validation. As shown in Figure 5A, the results of cross-validation suggested that the feature gene-based SVM classifier yield an accurate classifying of the discovery cohort between AMI and healthy controls with an AUC of 0.968 (95% confidence interval [CI]: 0.863–0.998), a sensitivity of 90.48% (95% CI: 69.6–98.8%), and a specificity of 95.45% (95% CI: 77.2–99.9%). The validation cohort was used to validate the performance of the SVM classifier in predicting AMI. The SVM classifier also demonstrated excellent discriminatory ability in this group with an AUC of 0.992 (95% CI: 0.922–1.00), a sensitivity of 96.43% (95% CI: 81.7–99.9%), and a specificity of 96.43% (95% CI: 81.7–99.9%; Figure 5B). Furthermore, the strong discrimination power was confirmed in the independent cohort with an AUC of 0.963 (95% CI: 0.870–0.996), a sensitivity of 90.32% (95% CI: 74.2–98.0%), a specificity of 90.48% (95% CI: 69.6–98.8%; Figure 5C). In addition, we explored the discrimination power of the classifier in terms of recurrent AMI. Surprisingly, the classifier presented outstanding discrimination ability for recurrent AMI with an AUC of 0.992 (95% CI: 0.874–1.00), a sensitivity of 100% (95% CI: 47.8–100.0%), and a specificity of 96.15% (95% CI: 80.4–99.9%; Figure 5D).

### Functional analysis of the feature ARGs

**Figure 6:**
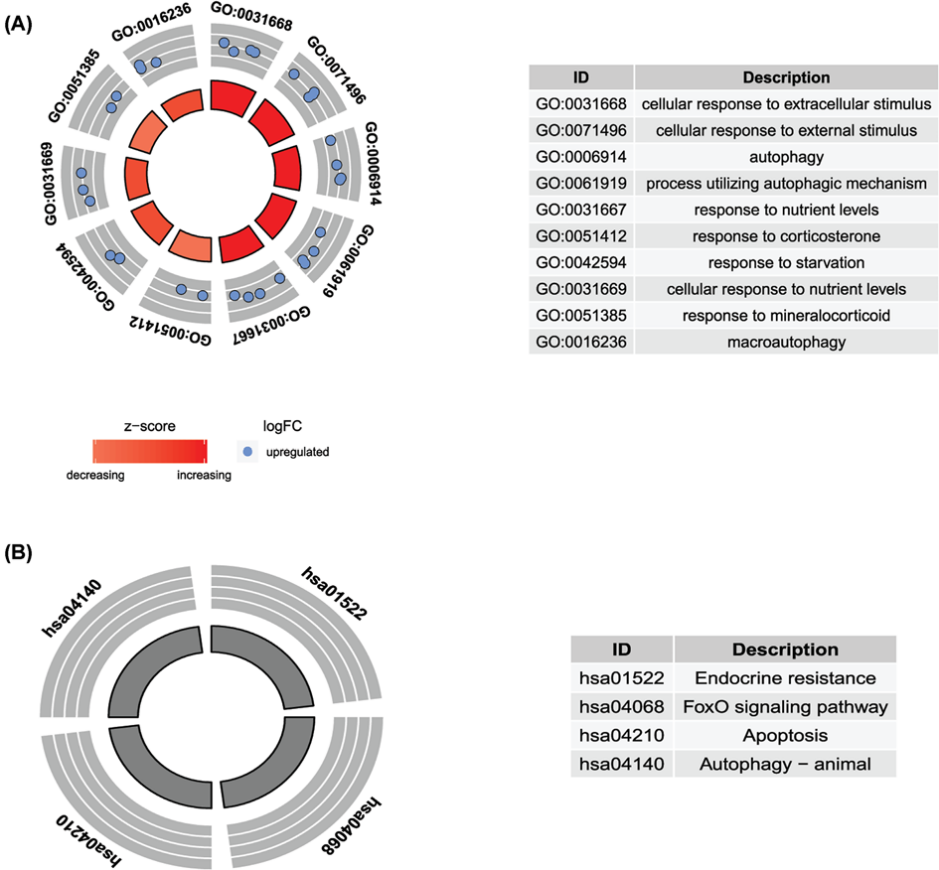
Gene ontology and Kyoto Encyclopedia of Genes and Genomes pathway analyses of the identified feature genes

To study the functional roles of the identified feature genes, GO enrichment, and KEGG pathway analyses were performed. Cellular responses to extracellular/external stimuli, autophagy, and macroautophagy were the most significantly enriched BPs (Figure 6A). In addition, apoptosis, autophagy, and the FoxO signaling pathway were considered to be the most remarkably enriched pathways (Figure 6B).

## Discussion

AMI is the primary cause of human mortality and morbidity worldwide [19]. The sensitivity and specificity of the existing biomarkers for AMI remain to be further elucidated. Recently, an association between autophagy and cardiac disorders such as myocardial infarction has been demonstrated [20]. However, numerous autophagy genes in AMI are not fully understood. The role of autophagy in diseases has received increasing attention from researchers. Therefore, to identify the ARGs in AMI, the discovery cohort (21 AMI samples and 22 healthy controls) from the GSE66360 dataset was used to screen differentially expressed genes in patient tissues compared with those in healthy tissues. Using the RFE method, the feature genes in AMI samples were identified, which allowed the AMI samples to be distinguished from the control samples. The identified feature genes were used to build an SVM classifier with an AUC of 0.968 for the patient samples. The discrimination power of the classifier for the validation cohort and the independent validation cohort was 0.992 and 0.963, respectively. Furthermore, the feature autophagy gene-based classifier successfully distinguished between recurrent AMI events and non-recurrent events with an AUC of 0.992. Therefore, the present study suggests that feature ARGs may be useful markers for identifying patients with AMI. In the original paper, 11 gene signatures (*HBEGF, SYTL3, EDN1, NR4A2, NFKBIA, VPS8, NR4A3, SULF1, RNASE1, CCL20*, and *MGP*) derived from gene expression profiling at the transcriptome level in whole blood were identified [21]. In our study, we identified seven feature ARGs in patients with AMI. There was no overlap between the ARGs and 11 gene signatures. The discrimination ability of the SVM classifier was slightly less than that of the original paper in terms of AUC. This is reasonable because the ARGs are parts of all the genes used in the original paper.

Our results demonstrated the potential value of the feature ARGs for the diagnosis of AMI in the clinical setting. *GABARAPL1, CDKN1A, DDIT3, NAMPT, FOS, WDFY3*, and *TP53INP2* were identified as potential genes associated with AMI and recurrence. *GABA* type A receptor associated protein like 1 (*GABARAPL1*) genes are located on human chromosome 12p12.3. They are involved in the proliferation, migration, angiogenesis, and autophagy of endothelial progenitor cells via regulation of miR-195 expression [22]. *GABARAPL1* has also been reported to be involved in autophagy signaling in myocardial infarction-induced muscle atrophy in rats [23]. The *CDKN1A* gene is a broad-acting cyclin-dependent kinase inhibitor that encodes the p21 protein [24]. In a Langendorff mouse model of hypoxia-reoxygenation myocardial injury, *Rev-Erbα* gene deletion or antagonist treatment weakened injury at the time of the sleep-to-wake transition by enhancing the expression of the ischemia–reperfusion injury modulator *CDKN1a/p21. CDKN1a/p21* was regarded as a downstream target of *Rev-Erbα* in the human myocardium, and *Rev-Erbα* protected cardiomyocytes from cell death [25,26]. *DDIT3* (DNA damage inducible transcript 3, also known as *CHOP*) is a key mediator of the endoplasmic reticulum stress response. It is considered to be a vital apoptotic signaling molecule after axonal injury [27]. Furthermore, *DDIT3* protein overexpression has been shown to induce apoptosis. Mammalian cell nicotinamide phosphoribosyl transferase (*NAMPT*) is the rate-limiting enzyme for nicotinamide adenine dinucleotide salvage synthesis in mammals. Increasing evidence suggests that *NAMPT* is involved in in metabolism and the immune response and plays pleiotropic roles in vascular homeostasis by regulating the functions of endothelial cells, vascular smooth muscle cells, endothelial progenitor cells, and perivascular cells [28,29]. Overexpression of *NAMPT* has been shown to enhance *SIRT1* activity, and protect cells from apoptosis via the activation of *SIRT3* and *SIRT4*. *NAMPT* can contribute to human vascular smooth muscle cell survival by activating *SIRT1* and preventing the accumulation of p53 [30]. Therefore, *NAMPT* may be an effective protective agent in the vasculature. *FOS*, also known as the proto-oncogene c-fos, has been demonstrated to be associated with apoptotic cell death. The high expression of c-fos may play a role in the pathogenesis of viral myocarditis, cardiac ischemia–reperfusion, myocardial stunning, and heart failure. For example, c-fos mRNA expression and protein expression rapidly increased at three days after coronary ligation in a rat model; furthermore, the infarct size was diminished in rats treated with c-fos monoclonal antibody compared with that of the control group [31]. This observation indicated that c-fos plays a vital role in myocardial lesions and is likely to be involved in the pathogenesis of AMI. As for *WDFY3* and *TP53INP2*, their roles in AMI have not been well studied and thus should be the focus of further research.

As revealed by the GO and KEGG pathway analyses, the identified ARGs were mainly involved in apoptosis, autophagy, and the FoxO signaling pathway. Autophagy is a physiological process for tissue survival. Apoptosis is a process of programmed cell death that naturally occurs in human body homeostasis and is known to be associated with myocardial ischemia [32]. The balance between autophagy and apoptosis determines cell survival and cardiac function, and the inflammatory response may be protective in the early stages of AMI via stimulation of myocyte autophagy [33]. However, it has also been noted that excessive autophagy may result in detrimental effects of cells [34]. Inflammation is an important regulator of autophagy. For example, *TNF-α* induce autophagy of cardiomyocytes *in vitro* and *in vivo* and contributes to autophagy by rapamycin protection against LPS-mediated myocyte apoptosis [35]. The FoxO signaling pathway has been reported to be involved in hypoxia injury in cardiac cells. *FOXO3* serves as a surveillance mechanism to detect and correct autophagy flux disruptions as well as ensure that the cells where autophagy is lacking are subjected to apoptosis [36]. Together, these finding and our results indicate that autophagy and ARGs play an important role in AMI.

However, we also recognize the shortcomings of the present study. First, we failed to validate the discriminatory ability of the SVM classifier in the independent patient cohort because of the lack of patient dataset with recurrent events. Second, there was an extremely significant difference in age distribution between AMI patients and control samples; thus, we were unable to adjust for the potential confounding effect in the present study. Furthermore, it should be noted that this research was based on bioinformatics analyses. Therefore, further validation from both *in vivo* and *in vitro* studies is needed.

In summary, *WDFY3, TP53INP2, GABARAPL1, CDKN1A, DDIT3, NAMPT*, and *FOS* are differentially expressed ARGs in AMI patients that may be used as potential biomarkers in the diagnosis of AMI. Identification of these ARGs helps to improve understanding of the molecular mechanism underlying AMI occurrence.

## Data Availability

The raw data of the present study are derived from the GEO data portal (https://www.ncbi.nlm.nih.gov/geo/; Accession number: GSE48060, GSE48060), which is a publicly available database.

## Abbreviations

AMI: acute myocardial infarction
ARG: autophagy-related gene
AUC: area under the curve
*DDIT3*: DNA damage inducible transcript 3
ECG: electrocardiogram
*NAMPT*: nicotinamide phosphoribosyl transferase
ROC: receiver operating characteristic
SVM-RFE: support vector machine-recursive feature elimination
